# Association of hOGG1 Ser326Cys, ITGA2 C807T, TNF-A -308G>A and XPD Lys751Gln polymorphisms with the survival of Malaysian NPC patients

**DOI:** 10.1371/journal.pone.0198332

**Published:** 2018-06-18

**Authors:** Eng-Zhuan Ban, Munn-Sann Lye, Pei Pei Chong, Yoke-Yeow Yap, Siew Ying Crystale Lim, Hejar Abdul Rahman

**Affiliations:** 1 Department of Community Health, Faculty of Medicine and Health Sciences, Universiti Putra Malaysia, Serdang, Malaysia; 2 Department of Biomedical Science, Faculty of Medicine and Health Sciences, Universiti Putra Malaysia, Serdang, Malaysia; 3 Department of Otorhinolaryngology, Faculty of Medicine and Health Sciences, Universiti Putra Malaysia, Serdang, Malaysia; 4 Faculty of Applied Sciences, UCSI University, Cheras, Malaysia; Chinese University of Hong Kong, HONG KONG

## Abstract

**Background:**

Nasopharyngeal carcinoma is a rare form of cancer across the world except in certain areas such as Southern China, Hong Kong and Malaysia. NPC is considered a relatively radiosensitive tumor and patients diagnosed at early stages tend to survive longer compared to those with advanced disease. Given that early symptoms of NPC are non-specific and that the nasopharynx is relatively inaccessible, less invasive screening methods such as biomarker screening might be the key to improve NPC survival and management. A number of genes with their respective polymorphisms have been shown in past studies to be associated with survival of various cancers. hOGG1 and XPD genes encode for a DNA glycosylase and a DNA helicase respectively; both are proteins that are involved in DNA repair. ITGA2 is the alpha subunit of the transmembrane receptor integrin and is mainly responsible for cell-cell and cell-extracellular matrix interaction. TNF-α is a cytokine that is released by immune cells during inflammation.

**Methods:**

Restriction fragment length polymorphism-polymerase chain reaction (RFLP-PCR) was used to genotype all the aforementioned gene polymorphisms. Kaplan-Meier survival function, log-rank test and Cox regression were used to investigate the effect of gene polymorphisms on the all-cause survival of NPC.

**Results:**

NPC cases carrying T/T genotype of ITGA2 C807T have poorer all-cause survival compared to those with C/C genotypes, with an adjusted HR of 2.06 (95% CI = 1.14–3.72) in individual model. The 5-year survival rate of C/C carriers was 55% compared to those with C/T and T/T where the survival rates were 50% and 43%, respectively.

**Conclusion:**

The finding from the present study showed that ITGA2 C807T polymorphism could be potentially useful as a prognostic biomarker for NPC. However, the prognostic value of ITGA2 C807T polymorphism has to be validated by well-designed further studies with larger patient numbers.

## Introduction

Nasopharyngeal carcinoma (NPC) is the commonest malignancy that originates from the nasopharynx. NPC is regarded as a rare form of cancer with varying disease prevalence across populations. The annual incidence of NPC in Southeast Asia [1], Southern China [2] and Hong Kong [3] is very high compared to the rest of the world. According to the International Agency for Research on Cancer, 84,400 NPC cases were newly diagnosed in 2008 with 51,600 deaths worldwide [4]. In Malaysia, NPC is the 4^th^ most common cancer [5]. In comparison with other carcinomas, NPC remains a relatively radiosensitive tumor [6]. Despite this, 20–30% of NPC patients still experience distant metastasis after combined intensity-modulated radiotherapy and chemotherapy [7]. Currently, tumor, node, metastasis (TNM) classification is mainly used for determination of prognosis and treatment strategy in NPC [8]. However, TNM staging is inadequate in the prognostic evaluation due to the heterogeneity of NPC, in which patients at the same TNM stage undergoing similar treatment modalities often experience different clinical outcomes [9]. Hence, development of a new prognostic marker is imperative for the improvement of the current management of patients diagnosed with NPC.

Integrins are transmembrane glycoprotein heterodimers that are made up from α- and β-subunits. So far 18 α- and 8 β-subunits have been discovered in mammals and they combine to form at least 25 different heterodimers that are specific for a unique set of ligands [10]. Integrins mainly serve as cell surface adhesive receptors that mediate cell-cell and cell-extracellular matrix interactions [11]. From previous research, integrins are shown to be involved in the regulation of cell proliferation, migration and survival [12–14]. Coupled with the downstream effect resulting from integrin signaling, integrin contributes to tumor progression through mediation of angiogenesis, lymphangiogenesis and inflammation in the tumor microenvironment [15–16]. Integrin α2 (ITGA2) from the α-family is a collagen receptor mainly expressed on platelets and epithelial cells [17]. ITGA2 807C>T (rs1126643) is one of the extensively studied polymorphisms of ITGA2. The expression of α2β1 integrin on the platelet surface has been reported to be lower in subjects carrying 807C compared to their 807T counterpart [18]. ITGA2 807C>T polymorphism was also found to be associated with the risk of developing breast and colorectal cancer [19–20].

Tumor necrosis factor alpha (TNF-α) is one of the earliest known cytokine produced in inflammatory process [21]. High circulating plasma level of TNF-α in patients with malignant lymphomas has been associated with poor disease outcome [22]. Excessive production is linked to weight loss, cachexia, anemia and modification of immune response reducing patients’ tolerance to their disease and treatment [23]. The TNF-α gene is located within class III region of major histocompatibility complex (MHC) [21]. A polymorphism located at position -308 that involves a base change of guanine to adenine was found to influence TNF expression in vitro [24]. Using human B-cell lines in transfection studies, the presence of TNF-α 308A allele was shown to correlate positively with higher inducible levels of TNF expression compared to 308G allele, indicating an important transcriptional regulation site is present in this region [25]. A allele of TNF-α -308G>A polymorphism was found in several studies to be associated with a decrease in survival of various cancers, namely B-cell lymphoma [26], non-Hodgkin lymphoma [27] and chronic lymphocytic lymphoma [28].

DNA repair is one of the important events in human cells that are responsible for maintaining the integrity of the human genome. DNA in human cells is constantly exposed to various endogenous and exogenous carcinogens from the surrounding environment [29]. Polycyclic aromatic hydrocarbons and nitrosamines are examples of exogenous carcinogens human cells are exposed to from ingestion of salted fish and cigarette smoking [30–31]. Normal cellular metabolic processes in the human body produce endogenous carcinogens such as hydroxyl radicals that can cause oxidative damage to DNA [32]. Accumulation of DNA damage, if unrepaired, leads to neoplastic transformation of normal cells [33]. Base excision repair (BER) and nucleotide excision repair (NER) are DNA repair pathways that are responsible of removing carcinogenic metabolites such as 8-oxo-7,8-dihydroguanine (8-oxoG) and bulky DNA adducts respectively [34–35]. Human 8-oxoguanine DNA N-glycosylase I (hOGG1) is the primary enzyme in BER pathway that recognizes 8-oxoG in the damaged DNA site [36]. Besides acting as a glycosylase, hOGG1 protein also function as an apurinic/apyrimidinic (AP) lyase that excises the damaged base from the DNA [37]. A polymorphism in codon 326 of hOGG1 protein has been shown to confer a higher risk of cancers, namely hepatocellular [38], breast [39] and orolaryngeal [40]. Similarly, hOGG1 Ser326Cys polymorphism was found to be significantly associated with survival of lung [41], bladder [42] and pancreatic cancer [43].

Xeroderma pigmentosum group D (XPD) gene on the other hand encodes for a 5’-3’ DNA helicase, which is an essential component in NER pathway [44]. XPD is responsible for unwinding the double helix DNA at the damaged site [45]. NER cannot proceed to the subsequent step of repair without the proper unwinding of DNA [46]. XPD Lys751Gln polymorphism is located within the carboxy-terminal domain (CTD) of XPD protein and is one of the established polymorphisms of the XPD gene [47]. Significant associations have been reported between XPD Lys751Gln polymorphism and several cancers, namely chronic myeloid leukemia [48] and esophageal [49] and nasopharyngeal carcinoma [50]. Studies on head and neck [51], lung [52], breast [53] and colorectal [54] cancers have shown significant association between XPD Lys751Gln polymorphism and survival.

In present study, we investigated the effect of gene polymorphisms on the overall and all-cause survival of NPC patients in Malaysia.

## Materials and methods

### Ethics statement and study design

This study was conducted with the approval from the Medical Research Ethics Committees of the Ministry of Health, Malaysia (NMRR-08-1572-3115). The present study was funded by Science Fund, Ministry of Science, Technology and Innovation (MOSTI; Project code: 04-11-08-625FR).

### Patient selection

NPC cases were selected from the NPC clinic registries in the Departments of Radiology and Oncology of two public hospitals starting in 2011. To minimize the incidence-prevalence bias [55], newly-diagnosed cases and cases diagnosed within two years from the time they were screened, were recruited. The inclusion criteria for NPC cases were NPC patients who were at least 18 years old and who have been confirmed histologically. Controls were age, sex and ethnicity matched to cases. Written informed consent was obtained from eligible cases prior to the enrollment into the study. The information sheet and consent form were available in both English and Malay. We assumed the exposure rate of ITGA2 C807T polymorphism at 49% from Chen et al [56], with two-sided alpha level of 0.05, 256 NPC cases were needed to attain a power of 90% to detect a 50% increase in hazard rate [57]. For the investigation of the all-cause survival, 300 NPC cases were available in the analysis.

### Endpoints

The primary endpoint was all-cause survival, defined as an estimate of the probability of surviving all-causes of death in a population. NPC patients who remained alive at the last follow-up were censored. In addition, 5-year overall survival rate was used as secondary endpoint, defined as the percentage of NPC patients who are alive at the end of 60 months from the time the disease was diagnosed.

### Sample preparation, DNA extraction and quantification of DNA yield

2.0 ml of fresh whole blood were collected from each study subject and were stored in EDTA tubes for preservation purposes. Cold chain (4°C) was maintained throughout the entire journey from hospitals to the laboratory in the university where the samples were processed. Genomic DNA was extracted from the blood samples using QIAamp® DNA Mini and Blood Mini kit (QIAgen, USA). The extracted DNA was stored at -80°C until further usage. The extracted DNA was quantified using nanophotometer and the absorbance was measured at wavelengths of 260nm and 280nm.

### DNA genotyping (RFLP-PCR)

hOGG1 Ser326Cys, ITGA2 C807T, TNF-α -308G>A and XPD Lys751Gln polymorphisms were assessed by using RFLP-PCR. The sequence of forward and reverse primers used for each polymorphism is tabulated in Table 1. For hOGG1 Ser326Cys polymorphism, the outcome of the PCR was a 302bp product. The composition of a total 25μl PCR reaction was 12.5μl of GoTaq® Green Master Mix (Promega, Wisconsin, USA), 0.5μl of each primer (from working concentration of 10 μm), 0.5μl of genomic DNA, and the remaining was topped up with nuclease free water. The PCR thermal profile used was initial denaturation at 95°C for 5 min, 32 cycles of 95°C for 30s, followed by 63°C for 30s and 72°C for 30s, then ended with final extension of 72°C for 5 min. PCR yield from previous PCR was then digested by the restriction enzyme *Fnu4HI* (New England Biolabs, Ipswich, USA). After the digestion, homozygous Ser/Ser showed only a single 302bp product while homozygous Cys/Cys was fully digested into two different products that were 186bp and 116bp in size. Samples were identified as heterozygous Ser/Cys if 3 products of different sizes appeared in the gel.

**Table 1:** Sequence of forward and reverse primers used in RFLP-PCR.

Polymorphisms	Forward primer (5’-3’)	Reverse primer (5’-3’)
hOGG1 Ser326Cys (rs1052133)	CTT CCA CCT CCC AAC ACT GTC AC	GTG CCT GGC CTT TGA GGT AGT C
ITGA2 C807T (rs1126643)	GTG TTT AAC TTG AAC ACA TAT	ACC TTG CAT ATT GAA TTG CTT
TNF-α -308G>A (rs1800629)	AGG CAA TAG GTT TTG AGG GCC AT	ACA CTC CCC ATC CTC CCT GCT C
XPD Lys751Gln (rs13181)	CCC CCT CTC CCT TTC CTC TG	AAC CAG GGC CAG GCA AGA C

For ITGA2 C807T polymorphism, 115bp product was generated at the end of the PCR protocol. The PCR composition used was 25μl PCR reaction consisting of 12.5μl of GoTaq® Green Master Mix (Promega, USA), 1.0μl of each primer (from working concentration of 10μm), 0.5μl of genomic DNA, and the remaining component was nuclease free water. The PCR thermal profile adopted was as follows: 95°C for 5 min, 35 cycles each of 95°C for 30s, followed by 55°C for 30s and 72°C for 30s, with final extension of 72°C for 5 min. The resulting PCR products were digested by restriction enzyme *TaqI* (New England Biolabs, Ipswich, US). Homozygous CC was fully digested into 2 products that were 92bp and 23bp in size after the excision by the restriction enzyme. For homozygous TT, no digestion occurred and only a single 115bp product was visible. All 3 products of different sizes were observed for heterozygous CT.

For TNF-α -308G>A polymorphism, 117bp PCR product spanning the promoter region of TNF-α from -238 to -308 was generated using the respective forward and reverse primers. The PCR composition used was 25μl PCR reaction consisting of 12.5μl of GoTaq® Green Master Mix (Promega, USA), 0.5μl of each primer (from working concentration of 10μm), 0.5μl of genomic DNA, and the remaining component was nuclease free water. The PCR thermal profile used was as follows: 95°C for 5 min; 95°C for 30s, 62°C for 30s and 72°C for 30s for 35 cycles; followed by 72°C for 5 min. Restriction enzyme *NcoI* (New England Biolabs, Ipswich, US) was utilized to excise the PCR products from the previous PCR run. Gel image of homozygous G/G showed only 2 products with 95bp and 22bp in size. Homozygous A/A was identified when the gel image showed a single product at 117bp while samples showing all 3 products were identified as heterozygous G/A.

For XPD Lys751Gln polymorphism, PCR product of 184bp in size was generated at the end of the PCR program. The PCR composition used was as follows: 25μl PCR reaction consisting of 12.5μl of GoTaq® Green Master Mix (Promega, USA), 0.5μl of each primer (from working concentration of 10μm), 0.5μl of genomic DNA, and the remaining was nuclease free water. The thermal profile of PCR used was 95°C for 5 min, 35 cycles each of 95°C for 40s, followed by 56°C for 30s and 72°C for 30s, with final extension of 72°C for 5 min. The resulting PCR products were digested by restriction enzyme *MboII* (New England Biolabs, Ipswich, US). Samples were identified as homozygous Lys/Lys if the results showed full digestion with only 2 products in size of 102bp and 82bp. Homozygous Gln/Gln samples showed only single PCR product that was 184bp in size. All 3 products of different sizes were observed for heterozygous Lys/Gln.

All digested PCR products were visualized under UV light after separation using electrophoresis on 3% ethidium bromide stained agarose gel. For quality control, 10% of the total PCR products were sent for DNA sequencing to confirm the results of RFLP-PCR for every polymorphism.

### Statistical analysis

The dependent variable was all-cause survival of NPC patients while independent variables were TNM staging, hOGG1 Ser326Cys, ITGA2 C807T, TNF-α -308G>A and XPD Lys751Gln polymorphisms. Potential confounders controlled in the present study were cigarette smoking, alcohol consumption, salted fish consumption during childhood. Relative frequencies, percentages (%), means and standard deviations were used to describe the characteristics of the studied population. All-cause survival was computed for TNM staging, hOGG1 Ser326Cys, ITGA2 C807T, TNF-α -308G>A and XPD Lys751Gln polymorphisms using Kaplan-Meier method while log-rank test was used to test the differences in survival between genotypes within each SNP. Cox proportional hazard model was used to obtain hazard ratios (HRs) and the respective 95% confidence intervals (CIs) adjusted for the aforementioned confounders, to identify potential role of polymorphism on the all-cause survival of NPC. The threshold for statistical significance was set at 0.05. All of the previously mentioned statistical analyses were performed using SPSS version 21.

## Results

### Characteristics of study population for NPC survival analysis

The median follow-up duration for all study participants was 46.1 months (range: 1.8–73.0). A total of 109 (36.3%) NPC cases died from all-causes during the present study while the remaining 191 (63.7%) cases were censored. From the 109 deaths, 41 (37.6%) were NPC-specific deaths while the other 68 (62.4%) NPC cases died from other causes. At time of diagnosis, 66 out of 287 (23.0%) NPC cases were primary tumor category T1 while 39.0% were category T2; the number of NPC patients diagnosed at categories T3 and T4 were 54 (18.8%) and 55 (19.2%), respectively. For lymph node category, the number of NPC cases diagnosed with N0 were 47 out of 287 (16.4%), those diagnosed with N1 were 76 (26.4%) in total while 117 (40.8%) NPC patients were diagnosed with N2 and the remaining 47 (16.4%) cases were diagnosed with N3. NPC patients that experienced metastasis comprised of 9 (3.1%) out of 288 cases while 279 (96.9%) did not experience metastasis. The socio-demographic characteristics, the distribution of cigarette smoking, alcohol consumption, salted fish consumption during childhood and genotype frequencies for hOGG1 Ser326Cys, ITGA2 C807T, TNF-α -308G>A and XPD Lys751Gln polymorphisms are shown in Table 2.

**Table 2:** Characteristics of NPC patients (N = 300).

Variables		
Median follow-up, months (range)		46.1 (1.8–73.0)
Age (Mean ± SD)		52.8 ± 10.9
		NPC cases, N = 300 (%)
Survival (all-cause)	Alive/Censored	191 (63.7)
	Death	109 (36.3)
Cause of death	NPC-specific	41 (37.6)
	Other causes	68 (62.4)
Gender (n = 300)	Male	232 (77.3)
	Female	68 (22.7)
Ethnicity (n = 300)	Malay	84 (28.0)
	Chinese	213 (71.0)
	Others	3 (1.0)
Salted fish consumption during childhood (n = 300)	Never	103 (34.3)
	Ever	197 (65.7)
Cigarette smoking (n = 300)	Never	146 (48.7)
	Ever	154 (51.3)
Alcohol consumption (n = 300)	Never	161 (53.7)
	Ever	139 (46.3)
TNM staging		
Primary tumor staging (T) (n = 287)	T1	66 (23.0)
	T2	112 (39.0)
	T3	54 (18.8)
	T4	55 (19.2)
Lymph node staging (N) (n = 287)	N0	47 (16.4)
	N1	76 (26.4)
	N2	117 (40.8)
	N3	47 (16.4)
Metastasis status (M) (n = 288)	M0	279 (96.9)
	M1	9 (3.1)
hOGG1 Ser326Cys (n = 300)	Ser/Ser	47 (15.7)
	Ser/Cys	161 (53.7)
	Cys/Cys	92 (30.6)
ITGA2 C807T (n = 300)	C/C	171 (57.0)
	C/T	103 (34.3)
	T/T	26 (8.7)
TNF-α -308G>A (n = 300)	G/G	244 (81.3)
	G/A+A/A	56 (18.7)
XPD Lys751Gln (n = 300)	Lys/Lys	256 (85.3)
	Lys/Gln+Gln/Gln	44 (14.7)

### Effect of gene polymorphisms and TNM staging on survival of NPC patients (univariate analysis)

Most of the aforementioned gene polymorphisms did not significantly influence the all-cause survival of NPC in univariate analysis (data not shown). The only significant difference was observed with ITGA2 C807 polymorphism; NPC patients carrying C/C genotype had a mean survival time of 57.8 months while those carrying C/T and T/T genotypes survived for an average of 53.5 and 46.2 months, respectively (*P*-value = 0.052, log-rank test).

Results from the univariate analysis showed that only metastasis status was significantly associated with the all-cause survival of NPC (Table 3). NPC cases who were diagnosed without metastasis had a mean survival time of 56.0 months compared with 32.3 months for those diagnosed with metastasis to other organs (*P*-value<0.01, log-rank test). For primary tumor and node categories, no significant difference in mean survival times was observed (*P*-value = 0.909 and 0.056, respectively, log-rank test).

**Table 3:** Effect of TNM staging on survival of NPC patients (univariate analysis, N = 300).

Variables		No of patients (%)	Mean survival time, months (95% CI)	p*-*value^a^
TNM staging				
Primary tumor staging (T) (n = 287)	T1	66 (23.0)	56.6 (51.0–62.1)	0.909
	T2	112 (39.0)	54.5 (49.9–59.0)	
	T3	54 (18.8)	55.2 (49.4–60.9)	
	T4	55 (19.2)	53.2 (46.6–59.8)	
Lymph node staging (N) (n = 287)	N0	47 (16.4)	52.9 (46.1–59.7)	0.056
	N1	76 (26.4)	53.6 (48.1–59.0)	
	N2	117 (40.8)	59.3 (55.3–63.3)	
	N3	47 (16.4)	55.2 (52.4–58.0)	
Metastasis status (M) (n = 288)	M0	279 (96.9)	56.0 (53.2–58.7)	0.002^b^
	M1	9 (3.1)	32.3 (17.6–47.0)	

^a^based on log-rank test

^b^p≤0.01

### Effect of individual gene polymorphisms and TNM staging on survival of NPC patients controlling for cigarette smoking, alcohol and salted fish consumption during childhood

**Fig 1:**
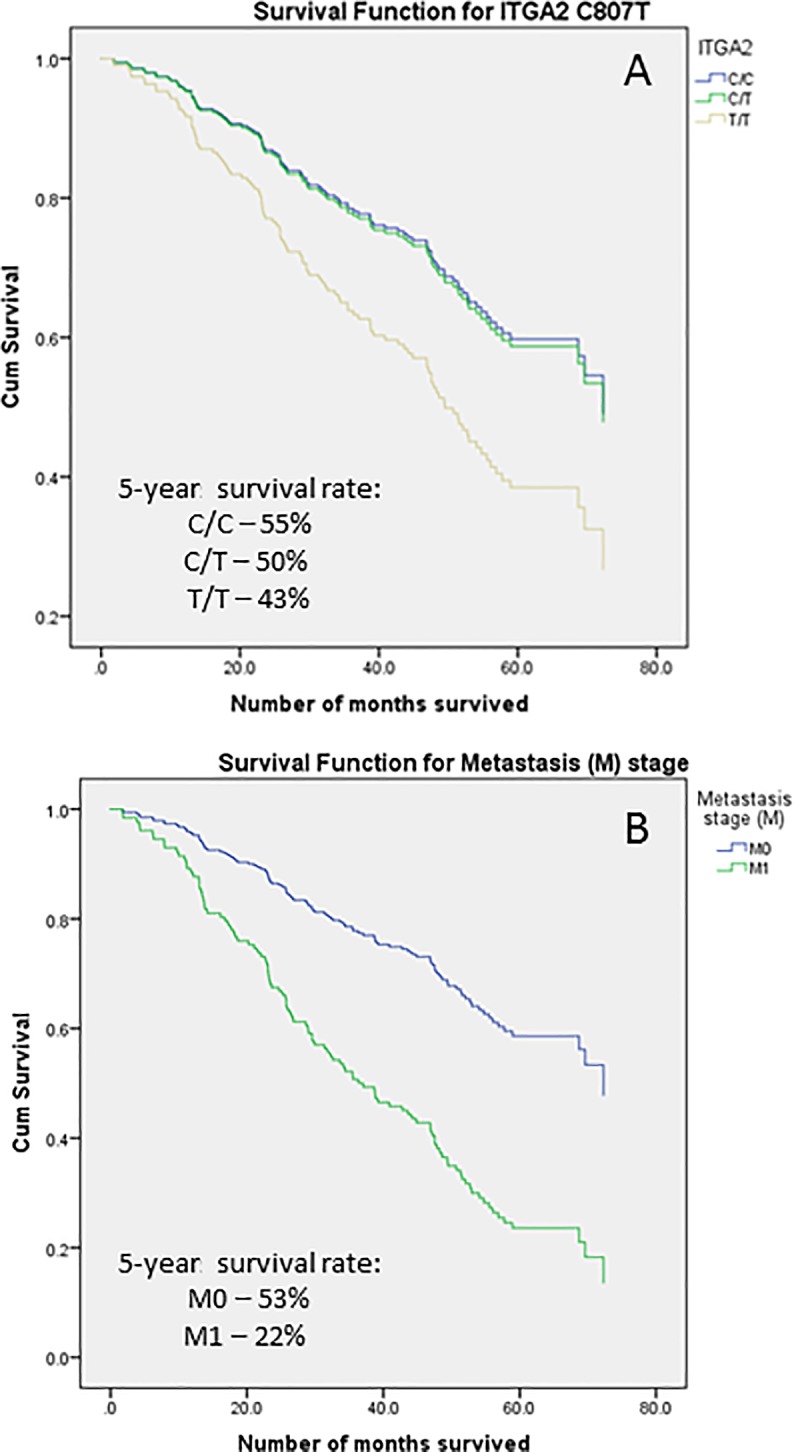
The survival functions for ITGA2 C807T polymorphism (A) and metastasis status (B).

Most of the variables mentioned above were not significantly associated with all-cause survival of NPC after adjusting for cigarette smoking, alcohol and salted fish consumption during childhood except for ITGA2 C807T polymorphism and metastasis status (Table 4). For ITGA2 C807T polymorphism, the adjusted HR for T/T genotype carriers was 2.06 (95% CI = 1.14–3.72) compared to the reference C/C genotype, while NPC cases with distant metastasis showed an adjusted HR of 3.13 (95% CI = 1.44–6.82) compared to those without metastasis, consistent with results from the log-rank test above. The survival functions for ITGA2 C807T polymorphism and metastasis status (M) are presented in Fig 1. The 5-year overall survival rate for ITGA2 C/C carriers was 55% compared to those with C/T and T/T genotypes in which the survival rates were 50% and 43%, respectively. NPC cases who experience distant metastasis had a 5-year overall survival rate of 22% compared to the survival rate of 53% for those without metastasis.

**Table 4:** Gene polymorphisms and TNM staging with the all-cause survival (N = 300) analyzed individually using Cox regression.

Variables		No of patients (%)	All-cause survival adjusted^a^ HR (95% CI)	p-value
hOGG1 Genotypes (n = 300)	Ser/Ser	47 (15.7)	1.0	-
	Ser/Cys	161 (53.7)	1.52 (0.84–2.76)	0.169
	Cys/Cys	92 (30.6)	1.22 (0.43–2.33)	0.548
ITGA2 Genotypes (n = 300)	C/C	171 (57.0)	1.0	-
	C/T	103 (34.3)	1.26 (0.84–1.89)	0.272
	T/T	26 (8.7)	2.06 (1.14–3.72)	0.017^b^
TNF-α Genotypes (n = 300)	G/G	244 (81.3)	1.0	-
	G/A + A/A	56 (18.7)	1.01 (0.63–1.64)	0.957
XPD Genotypes (n = 300)	Lys/Gln + Gln/Gln	44 (14.7)	1.0	-
	Lys/Lys	256 (85.3)	1.15 (0.67–1.99)	0.614
TNM staging				
Primary tumor staging (T) (n = 287)	T1	66 (23.0)	1.0	-
	T2	112 (39.0)	1.08 (0.64–1.81)	0.785
	T3	54 (18.8)	0.93 (0.50–1.73)	0.824
	T4	55 (19.2)	1.20 (0.66–2.16)	0.557
Lymph node staging (N) (n = 287)	N0	47 (16.4)	1.0	-
	N1	76 (26.4)	1.06 (0.59–1.89)	0.858
	N2	117 (40.8)	0.69 (0.39–1.22)	0.199
	N3	47 (16.4)	1.36 (0.73–2.51)	0.331
Metastasis status (M) (n = 288)	M0	279 (96.9)	1.0	-
	M1	9 (3.1)	3.13 (1.44–6.82)	0.004^b^

^a^hazard ratio adjusted for salted fish consumption during childhood, cigarette smoking, alcohol consumption.

^b^p≤0.05

## Discussion

The distribution of TNM staging in the present study was mostly comparable to that of a number of studies from different populations. The only exception was that the proportion of NPC cases diagnosed with T1 staging was higher in the present study compared studies done elsewhere [58–60].

NPC cases carrying T/T genotype of ITGA2 C807T were observed to have a poorer all-cause survival as compared to those with C/C genotypes, with an adjusted HR of 2.06 (95% CI = 1.14–3.72) in individual model. The 5-year survival rate of C/C carriers was 55% compared to those with C/T and T/T where the survival rates were 50% and 43%, respectively. Our results also showed that NPC patients with ITGA2 C807T T/T genotype (3.8% metastasized) was more susceptible to metastasis compared to their C/C counterparts (1.8% metastasized). To the best of our knowledge, this is the first report of a significant association between integrin α2 polymorphism and survival in NPC. Molecular studies on human cancer have shown that tumor cells switch their integrin expression based on the stage of progression of the disease [61–62]. As a consequence of the changes in integrin expression, neoplastic cells tend to lose the integrins that maintain their adhesion to the basement membrane, at the same time, overexpressing the integrins that foster tumor cells’ survival, migration and proliferation [63]. In addition, integrins were found to provide the anchorage necessary for tumor cells’ migration during metastasis through activation of pro-migratory signals. Invasive malignant cells were found to be constantly making and breaking integrin contacts during cell migration [63]. Activation of myosin II by integrin-induced ERK pathway generates the contractile force that pulls the cells forward along the actin cables towards the newly-formed integrin contacts and breaks the adhesion at the trailing edge of the cells [64].

Integrin α2 is a collagen receptor that is mainly expressed on platelets as well as epithelial cells and the polymorphism in this study is located on position 807 was implicated in several cancer studies to be associated with increased cancer risk [19–20, 65]. Loss of ITGA2 expression was also found to be associated with metastasis of breast and colon cancer [17, 66]. Re-expression of α2β1 in breast cancer cells was shown to reverse the tumorigenic properties of the cells [67]. Similar results has been found in an animal study where integrin α2 was observed to be a metastasis suppressor as lack of α2β1 expression resulted in markedly increased cancer metastasis [68]. Deletion of α2β1 expression was also found in the same study to enhance tumor intravasation [68]. Suppression of FAK-CD-mediated loss of adhesion was also observed when integrin α2 was overexpressed due to activated Src in breast cancer cells [69]. In addition to its role in suppressing metastasis, integrin α2 causes apoptotic cell death via α2β1 transducing the necessary signal that promote cell death in matrix deprived cells [70].

However, contradicting results were found as integrin α2 has been linked to increased adhesion and migration facilitated by MAPK in an in-vivo study involving breast cancer cell [71]. In addition, integrin α2β1 was shown to promote prostate cancer cell metastasis to bone [72]. Inconsistent results from various cancer studies on the role of integrin α2 in cancer progression may suggest that integrin’s function is cell type and context dependent. Such a hypothesis is supported by a finding showing that different responses have been observed when integrin α2 knockout mice were challenged with 2 different cancer cells, namely B16F10 melanoma cells and Lewis Lung carcinoma cells [73]. Challenging the integrin α2 knockout mice with the former cell type resulted in increased tumor angiogenesis and yet there was no response from challenge of the latter [73].

Based on the results from the present study, it is postulated that altered expression of ITGA2 arising from the polymorphic difference of C807T resulted in increased tumor cell metastasis and intravasation. Apoptotic cell death resulting from the detachment of cells from the surrounding ECM might be suppressed as signal transduced by α2β1 integrin is required for the process. Thus, the effect of ITGA2 C807T polymorphism on both metastasis and apoptotic cell death might be one of the key contributing factors for shorter survival of NPC cases carrying the susceptible 807 T/T genotype compared to those with C/C genotype.

There is the question regarding the usefulness of ITGA2 C807T polymorphism in prognosticating the survival of NPC patients if only 8.7% of NPC patients carry the genotype associated with better survival. Given the current magnitude of percentage, the application of ITGA2 C807T in a mass screening program for prognostication of NPC patients’ survival would not be feasible. However, if other researchers can replicate and validate findings from the present study, a sizable number of NPC patients would have benefitted from the accurate prognostic prediction. ITGA2 C807T might be more clinically useful if it is used as a supplementary test for targeted populations on a limited basis.

## Conclusion

The finding from the present study showed that ITGA2 C807T polymorphism could be potentially useful as a prognostic biomarker for NPC. NPC patients having T/T genotype were found with poorer 5-year survival compared to the wildtype C/C carriers. If ITGA2 C807T polymorphism is verified as a valid prognostic marker for NPC patients, the possibility exists for customizing treatment modalities for individuals with ITGA2 C807 T/T genotype in an attempt to enhance survival. However, the prognostic value of ITGA2 C807T polymorphism has to be validated by well-designed further studies with larger patient numbers.

## Supporting information

S1 Appendix Representative RFLP-PCR gel image and sequencing chromatogram for hOGG1 Ser326Cys polymorphism. (TIF)

S2 Appendix Representative RFLP-PCR gel image and sequencing chromatogram for ITGA2 C807T polymorphism. (TIF)

S3 Appendix Representative RFLP-PCR gel image and sequencing chromatogram for TNF-α -308G>A polymorphism. (TIF)

S4 Appendix Representative RFLP-PCR gel image and sequencing chromatogram for XPD Lys751Gln polymorphism. (TIF)
