# Supplementary figures and images for: Association of hOGG1 Ser326Cys, ITGA2 C807T, TNF-A -308G>A and XPD Lys751Gln polymorphisms with the survival of Malaysian NPC patients
Source: PLoS One. 2018 Jun 18;13(6):e0198332. doi: 10.1371/journal.pone.0198332 (PMC6005472; doi:10.1371/journal.pone.0198332)

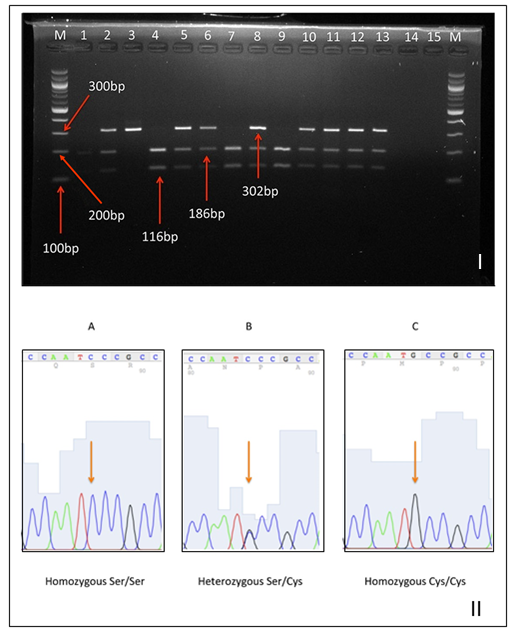

Supplement: S1 Appendix — (TIF) [file pone.0198332.s001.tif]

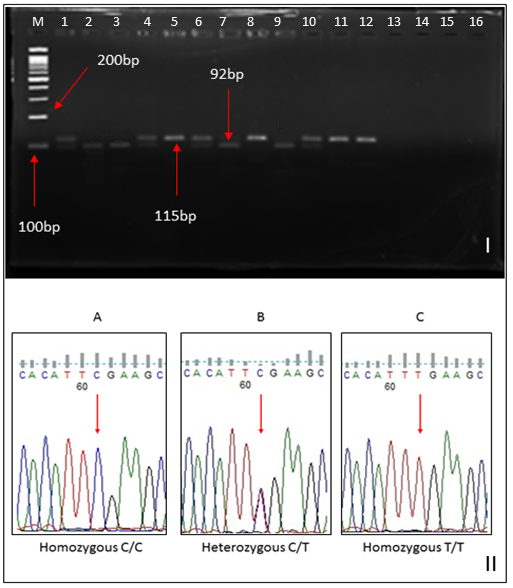

Supplement: S2 Appendix — (TIF) [file pone.0198332.s002.tif]

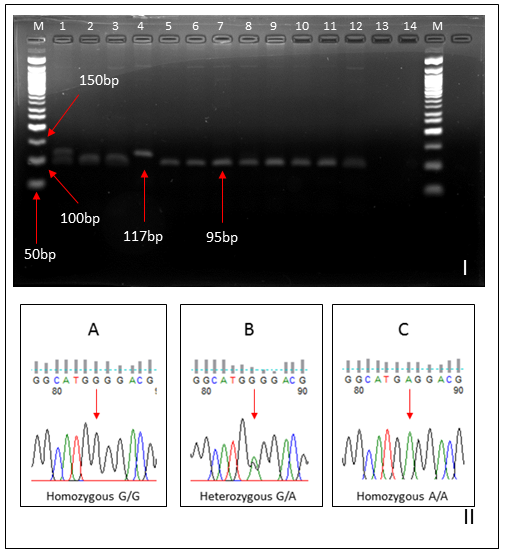

Supplement: S3 Appendix — (TIF) [file pone.0198332.s003.tif]

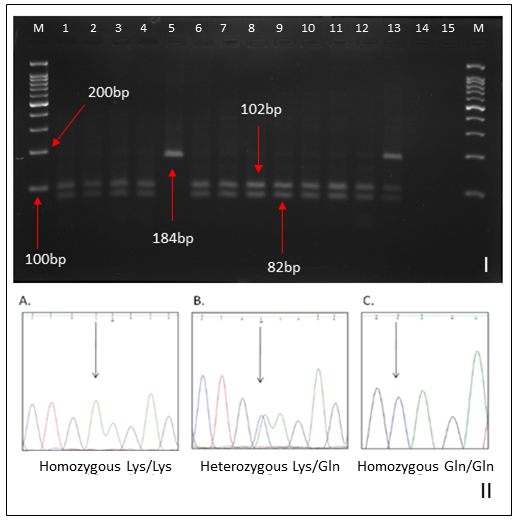

Supplement: S4 Appendix — (TIF) [file pone.0198332.s004.tif]
